# Residual intraperitoneal carbon dioxide gas following laparoscopy for adnexal masses: Residual gas volume assessment and postoperative outcome analysis

**DOI:** 10.1097/MD.0000000000030142

**Published:** 2022-09-02

**Authors:** Sang Wook Yi

**Affiliations:** a Division of Minimally Invasive Surgery and Gynecological Laparoendoscopy, Department of Obstetrics and Gynecology, Gangneung Asan Hospital, University of Ulsan College of Medicine, Gangwon, South Korea.

**Keywords:** adnexa, drainage tube, laparoscopy, shoulder pain, single-port laparoscopy, volume of residual carbon dioxide

## Abstract

Free residual gas after laparoscopy may cause shoulder pain, decreasing patient satisfaction with the procedure. We analyzed the correlation between postoperative residual carbon dioxide gas and shoulder pain, explored the peri- and postoperative factors associated with residual carbon dioxide and determined the effects of the use of a drainage tube. A cohort of 326 patients who underwent laparoscopic adnexal surgery between March 2005 and June 2018 at a teaching hospital in Korea was retrospectively analyzed through a medical records review. The enrolled patients were divided into 1-, 2-, and 3-port groups. The right volume, left volume, and total volume of residual gas were calculated using a formula based on measurements obtained from chest X-rays. Continuous variables were compared using Student *t* tests. Categorical variables were compared with the chi-square test or Kruskal–Wallis test. The total volumes of postoperative residual carbon dioxide gas were significantly different between the 1- and 2-port groups and between the 1- and 3-port groups (157.3 ± 179.2 vs 25.1 ± 92.3 mL and 157.3 ± 179.2 vs 12.9 ± 36.4 mL, respectively). The volume of residual gas and the time to the first passage of gas were positively correlated. The total volume of residual gas was more strongly correlated with the operative wound pain score than with the shoulder pain score. Additionally, the pre- and postoperative white blood cell counts, postoperative hospitalization duration, residual carbon dioxide volume, and shoulder pain score were significantly different between patients with and without a drainage tube. Although the volume of residual gas was not correlated with the shoulder pain score, the author found that both were lower in patients with a drainage tube than in those without, indicating that a drainage tube could be safely used to decrease residual gas volume and the shoulder pain score without increasing the risk of postoperative infection.

## 1. Introduction

To perform laparoscopy, pneumoperitoneum must be induced by inserting carbon dioxide gas into the peritoneal cavity. Although many surgeons attempt to remove residual carbon dioxide gas from the peritoneal cavity after laparoscopic procedures, postoperatively, a large volume of residual carbon dioxide remains as free gas.^[1]^ Postoperative residual carbon dioxide gas tends to accumulate below the diaphragm and around the liver and stomach due to the density of gas and the effects of gravity. Free residual carbon dioxide gas is thought to cause shoulder pain and upper abdominal pain in patients by irritating the phrenic nerve.^[1]^ However, the causes of postoperative shoulder pain are unknown, and the correlation between the amount of free air and shoulder pain is also uncertain. Some authors reported that all patients began to get out of bed on the first day (12–24 hours) after surgery. Most of the patients began to have shoulder pain after getting out of bed for the first time. It may be that the location of gas accumulation in the abdominal cavity changes with body position and then causes shoulder pain.^[2]^ Although the definite cause of shoulder pain after laparoscopy is unknown, free gas after laparoscopy in the abdominal cavity is suggested to be correlated with shoulder pain. With this, some authors have tried to decrease shoulder pain in patients with suction drains or low flow rates to induce pneumoperitoneum followed by high flow rates.^[3–5]^

Many surgeons neglect to remove free residual gas because the shoulder pain it causes tends to resolve spontaneously within several days. Some patients who undergo laparoscopy complain that they have more shoulder pain than operative wound pain, and this pain may decrease a patient’s satisfaction with the procedure.^[2,6–8]^ Because most patients think shoulder pain has nothing to do with surgery, it makes them more anxious. This may lead to discomfort and poor quality of life after laparoscopic surgery and greatly reduce patient satisfaction.

Recently, advancements in minimally invasive procedures have allowed 1-port laparoscopy to be performed in many hospitals. Single-port laparoscopy has some merits, including better cosmetic results, because these surgical procedures leave only 1 operative wound scar in the umbilicus.^[9]^ Aside from its cosmetic merit, patients who undergo 1-port laparoscopy have shown similar levels of postoperative shoulder pain to those treated with conventional laparoscopy.^[6,10]^ The factors that cause shoulder pain and the perioperative and postoperative factors associated with shoulder pain or residual gas need to be explored.

The author therefore sought to analyze the correlation between the volume of postoperative residual carbon dioxide gas and postoperative shoulder pain and explore the perioperative and postoperative factors associated with the 2. The correlation between postoperative pain and postoperative shoulder pain after laparoscopy was assessed to explore the cause of shoulder pain. Additionally, the author suggests a strategy for decreasing the volume of residual carbon dioxide gas.

## 2. Materials and Methods

A retrospective cohort study was performed via a review of the medical records and radiological studies related to patients who underwent laparoscopic adnexal surgery at a teaching hospital in Korea between March 2005 and June 2018. This study was approved by the institutional review board of our hospital (GNAH2017-09-001). Of the 358 patients who underwent laparoscopy for adnexal surgery during the study period, 326 were enrolled, and 32 patients were excluded for not having undergone a postoperative chest X-ray examination, as it is not a standard procedure and is performed only if the patient experiences shoulder pain or respiratory symptoms. Because many patients who underwent laparoscopy had complaints of shoulder pain or dyspnea, chest X-rays in the upright position were examined in the radiology room for residual carbon dioxide or other lung lesions on postoperative day 2. Because this was a retrospective cohort study, patients who did not undergo chest X-ray were excluded from this study because the residual carbon dioxide volume was calculated from chest X-ray.

**Figure 1.**
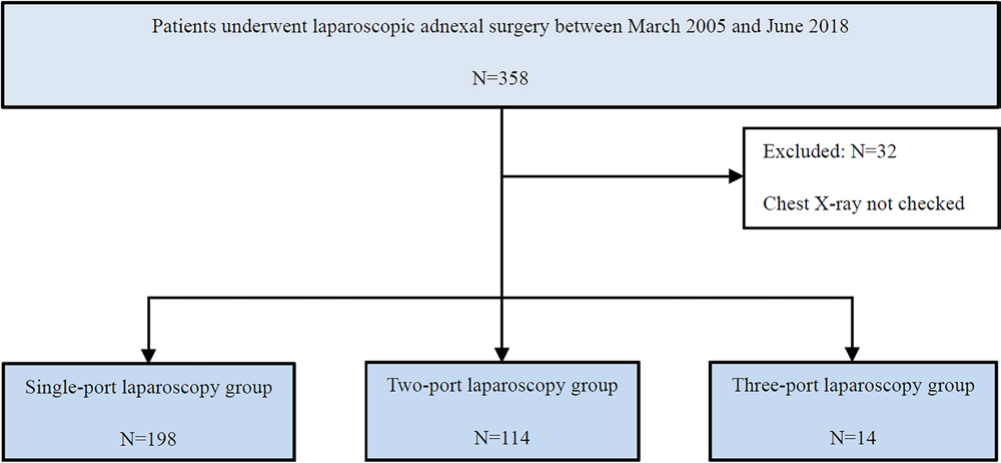
The flow diagram of this study.

The enrolled patients were a consecutive case group and divided into 3 study groups according to the number of ports used into the 1-port (198 patients), 2-port (114 patients), and 3-port (14 patients) groups (Fig. 1).

### 2.1. Preoperative preparation

Patients were admitted the day before surgery, and consent for treatment was obtained from all patients. The possibility of conversion to laparotomy in cases of severe adhesion, malignancy, or inadequate visualization of the operative field was explained. A Fleet enema was administered at 7:00 pm to evacuate the lower bowel. The laparoscopic procedure was performed under general anesthesia with endotracheal intubation and placement of an orogastric tube. Patients were placed in the lithotomy position with their arms at their sides to enable the use of a uterine manipulator or in the supine position if they were young with no history of coitus. One dose of prophylactic antibiotics was administered before anesthesia was induced. A Foley catheter was inserted into the urethra. A Kronner Manipujector^®^ uterine manipulator (Cooper Surgical, Trumbull, CT) was inserted into the uterine cavity, except in young patients with no history of coitus.

Because a single surgeon performed the procedures for all patients enrolled in this study, the surgical procedures were performed using the same methods. A small longitudinal skin incision was made in the umbilicus, and a Veress needle was inserted to establish pneumoperitoneum. A 10-mm trocar was then placed in the umbilical area, and a 10-mm, 0° laparoscope was inserted through the trocar. The pelvic anatomy was carefully inspected to determine whether conversion to laparotomy was necessary.

### 2.2. Port preparation in 1- and 2-port laparoscopic adnexal surgery

In 1-port laparoscopic surgeries, 3 trocars (two 12-mm trocars and one 5-mm trocar) were inserted into separate fingers of a surgical glove and secured with rubber bands. The wrist portion of the glove covered the wound retractor, and 3 Babcock clamps were placed on the edges of the retractor to prevent carbon dioxide leakage. A 10-mm laparoscope and atraumatic forceps were inserted through the umbilical multichannel port.

In 2-port laparoscopic surgeries, an ancillary 5-mm trocar was placed low in the left abdomen under laparoscopy. The umbilical trocar was removed, and the skin incision was extended to approximately 1.5 cm, which is sufficiently wide to allow passage of an index finger. The skin incision was extended to the upper and lower margins of the umbilicus to minimize abdominal scarring. An extrasmall Alexis^®^ wound retractor (Applied Medical, Rancho Santa Margarita, CA) was placed in the umbilical incision. Two 12-mm trocars were inserted into separate fingers of a No. 6 surgical glove and secured with rubber bands, and the other 3 fingers of the glove were tied together.^[11]^

### 2.3. Port preparation in 3-port laparoscopic surgery

Ancillary 12-, 10-, or 5-mm trocars were inserted low in the right and left abdomen under laparoscopic observation. In the conventional laparoscopy group, the ancillary port site low in the abdomen was extended to remove surgical specimens, if necessary. A wound retractor was not used in the 3-port laparoscopic procedure.

### 2.4. Adnexal surgery procedure

The pelvic masses were primarily ovarian cysts and tumors. The procedures performed included adnexectomy, cystectomy, and myomectomy in 1 case where a pedunculated subserosal myoma had been initially diagnosed as an adnexal mass. A Jackson–Pratt-style drainage tube (Barovac^®^, Sejong Medical, Co., Ltd., Korea) was placed through the left 5-mm port site if inflammation, adhesion, or hemorrhage was evident, and the abdominal wounds were sutured.

The surgeon did not perform 1-port laparoscopy in patients with inflammation, severe adhesions, or a tendency to hemorrhage and did not insert drainage tubes in patients who underwent 1-port laparoscopy due to concerns about umbilical wound discharge and wound problems after drainage tube removal. Therefore, the drainage tube was used only after 2- or 3-port laparoscopy for adnexal surgery in the enrolled patients.

### 2.5. Outcome measurements

Following a review of the medical records and radiological studies, the clinicopathological characteristics of the patients, such as age, parity, and previous medical and surgical histories, and operative outcomes, such as the operative time, pre- and postoperative white blood cell (WBC) counts, change in the hemoglobin (Hb) level, time to the first passage of gas, postoperative hospitalization duration, postoperative shoulder pain score and operative wound pain score, were investigated. Usually, in our hospital, the postoperative pain score assessment has been included in postoperative nursing care. With this, all postoperative patients were assessed for postoperative shoulder and operative wound pain scores according to a verbal rating scale (VRS; pain scores: 0–10) every 4 hours for a total of 10 times or more. However, missing pain scores were analyzed as a missed value.

**Figure 2.**
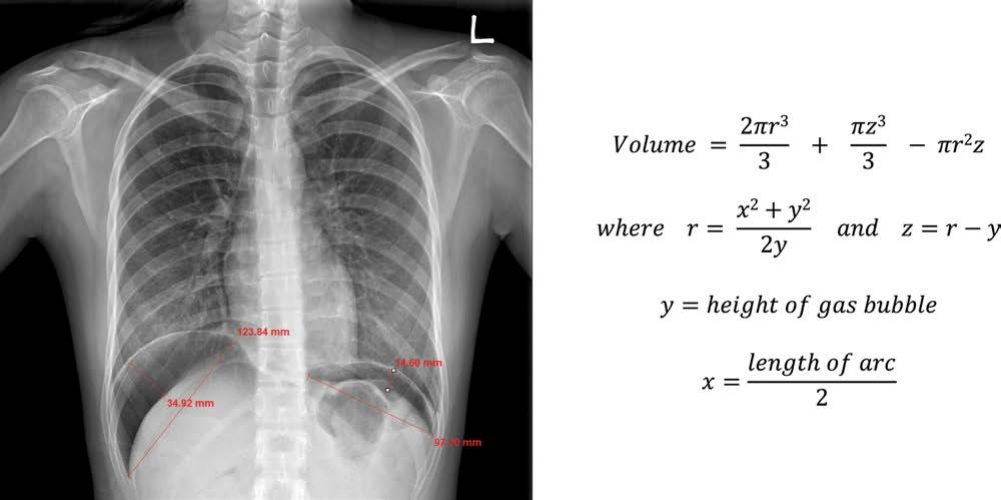
Chest X-ray (left) showing large gas bubbles under each hemidiaphragm. Dimensions on the right: length of arc, 12.4 cm; height, 3.5 cm; volume, 233.7 mL. Dimensions on the left: length of arc, 9.8 cm; height, 1.5 cm; volume, 58.3 mL. All volumes were calculated using the formulas shown on the right.

The volume of residual carbon dioxide gas was measured as the right volume, the left volume, and the total volume of residual gas using a formula that incorporated measurements obtained on chest X-ray (Fig. 2). On chest X-ray, the length of the arcs and the height of the gas bubbles on the right and left sides were measured using the adjusted scale on the X-ray films^[12]^ to calculate the volumes with the respective formulas. The calculated volumes of residual gas on the right and left sides were added together to obtain the total volume of residual carbon dioxide gas.

### 2.6. Statistical analysis

The results were analyzed using SPSS^®^ statistical software (version 10.0 for Windows; SPSS, Inc., Chicago, IL). Continuous variables were compared using Student *t* test. Categorical variables were compared with the chi-square or Kruskal–Wallis test. All tests were 2-sided, and a *P* value of ≤.05 was regarded as significant.

## 3. Results

Analysis of the clinical characteristics in the 1-, 2-, and 3-port groups showed that the mean patient ages were 34.6 ± 12.1, 35.4 ± 12.4, and 29.6 ± 10.2 years, respectively. There were no significant differences in the mean patient age among the study groups. In the 1-, 2-, and 3-port groups, the mean parity was 1.1 ± 1.2, 1.0 ± 1.1, and 0.4 ± 0.8, respectively, with no significant differences among the groups. There were also no significant differences in height, body weight, or body mass index (BMI) among the study groups (Table 1).

**Table 1:** Clinical characteristics of the study groups.

	1-port group (N = 198)	2-port group (N = 114)	3-port group (N = 14)	*P* value
Age (yr)	34.6 ± 12.1	35.4 ± 12.4	29.7 ± 10.2	.143
Parity	1.1 ± 1.2	1.0 ± 1.1	0.4 ± 0.8	.109
Height (cm)	159.7 ± 5.8	159.9 ± 6.6	161.6 ± 5.9	.549
Weight (kg)	59.8 ± 11.7	59.9 ± 13.7	55.3 ± 8.6	.339
BMI (kg/m^2^)	23.5 ± 4.3	24.6 ± 13.9	21.2 ± 3.0	.161

BMI = body mass index.

A comparison of the peri- and postoperative surgical outcomes revealed that the operative time, time to the first passage of gas, and postoperative shoulder pain score were not significantly different among the study groups. However, the pre- and postoperative WBC counts, change in the Hb level, postoperative hospitalization duration and total volume of postoperative residual carbon dioxide gas were significantly different among the study groups (Table 2).

**Table 2:** Surgical outcomes of the study groups.

	1-port group	2-port group	3-port group	*P* value
Operative time (min)	78.7 ± 21.5	76.1 ± 22.2	69.3 ± 39.3	.170
Preoperative WBC	6360.1 ± 1923.2	9200.9 ± 4600.4	8971.4 ± 6249.6	.000*
Postoperative WBC	6869.2 ± 1804.5	6678.1 ± 2288.9	5421.4 ± 1678.0	.007†
Hb change (g/dL)	1.7 ± 0.8	1.8 ± 1.1	2.7 ± 1.0	.001‡
Time to first passage of gas (h)	43.7 ± 16.4	41.3 ± 16.8	30.9 ± 1.0	.149
Postoperative hospitalization duration (d)	3.88 ± 0.7	4.4 ± 1.0	4.9 ± 1.1	.000§
Total residual CO_2_ volume (mL)	157.3 ± 179.2	25.1 ± 92.3	12.9 ± 36.4	.000∥
Shoulder pain score	2.1 ± 1.9	1.3 ± 1.0	2.0 ± 1.4	.362

Hb = hemoglobin, WBC = white blood cell.

* 1-port vs 2-port *P* = .000; 2-port vs 3-port *P* = .289; 1-port vs 3-port *P* = .509.

† 1-port vs 2-port *P* = .135; 2-port vs 3-port *P* = .028; 1-port vs 3-port *P* = .002.

‡ 1-port vs 2-port *P* = .077; 2-port vs 3-port *P* = .007; 1-port vs 3-port *P* = .000.

§ 1-port vs 2-port *P* = .000; 2-port vs 3-port *P* = .027; 1-port vs 3-port *P* = .000.

∥ 1-port vs 2-port *P* = .000; 2-port vs 3-port *P* = .097; 1-port vs 3-port *P* = .000.

The preoperative WBC count was significantly different between the 1- and 2-port groups (6360.1 ± 1923.2 vs 9200.9 ± 4600.4 cells/µL), the 1- and 3-port groups (6869.2 ± 1804.5 vs 5421.4 ± 1678.0 cells/µL), and the 2- and 3-port groups (6678.1 ± 2288.9 vs 5421.4 ± 1678.0 cells/µL). The change in Hb level was significantly different between the 1- and 3-port groups and between the 2- and 3-port groups (1.7 ± 0.8 vs 2.7 ± 1.0 g/dL and 1.8 ± 1.1 vs 2.7 ± 1.1 g/dL, respectively). The postoperative hospitalization duration was significantly different between the 1- and 2-port groups, the 2- and 3-port groups, and the 1- and 3-port groups (3.9 ± 0.7 vs 4.4 ± 1.0 days; 4.4 ± 1.0 vs 4.9 ± 1.1 days; and 3.9 ± 0.7 vs 4.9 ± 1.1 days, respectively).

The total volume of postoperative residual carbon dioxide gas was significantly different between the 1- and 2-port groups and the 1- and 3-port groups (157.3 ± 179.2 vs 25.1 ± 92.3 mL and 157.3 ± 179.2 vs 12.9 ± 36.4 mL, respectively). However, the difference between the 2- and 3-port groups was not significant.

There were correlations between the total volume of postoperative residual carbon dioxide and weight, BMI, and port number, with heavier and more obese patients having more residual carbon dioxide gas and patients in whom more ports were used having less residual carbon dioxide gas.

With respect to the number of ports used, there was no difference in the postoperative volume of residual carbon dioxide gas between the 2- and 3-port groups. A drainage tube was used in all patients treated with 2-port and 3-port laparoscopy and played a major role in decreasing the volume of postoperative residual carbon dioxide gas.

There was no correlation between the total volume of postoperative residual carbon dioxide and operation time (Table 3). The volume of residual gas and the time to the first passage of gas were positively correlated. Therefore, patients with more residual carbon dioxide tended to require more postoperative time for the first passage of gas to occur. The volume of residual carbon dioxide gas was negatively correlated with postoperative hospitalization duration. However, the postoperative hospitalization duration was 4 to 5 days for patients without any postoperative complications and did not change according to the time to the first passage of gas. Because the postoperative hospitalization duration was shorter in the 1-port group than in the 2- or 3-port group, the total volume of residual carbon dioxide gas was higher in the 1-port group and was correlated with the postoperative hospitalization duration. In this study, the postoperative hospitalization duration was longer not only due to postoperative complications but also based on patient preference. Therefore, the correlation between the volume of residual gas and postoperative hospitalization duration may not be meaningful. The postoperative hospitalization duration for patients in this study was longer than that of patients in hospitals in other countries. In Korea, patients tend to want to stay in the hospital for 4 or 5 days after an operation, which may be due to the relatively low medical expenses in Korea and patient anxiety regarding short hospital stays. For these reasons, most patients were not discharged if they did not want to leave.

**Table 3:** Correlations between intra-abdominal residual carbon dioxide volume and clinical factors.

	Pearson correlation	*P* value
Operative time (min)	−0.004	.947
Hb change (g/dL)*	−0.002	.966
Time to first passage of gas (h)	0.146	.009†
Postoperative hospitalization duration (d)	−0.119	.031†
Shoulder pain score of 2	0.230	.043†
Operative wound pain score of 3	−0.114	.048†
Operative wound pain score of 10	−0.230	.000†

Hb = hemoglobin.

* Hemoglobin change is shown as the difference between preoperative hemoglobin and 2-d postoperative hemoglobin.

† *P* < .05, significant.

The postoperative shoulder and operative wound pain scores were assessed according to a VRS (pain scores: 0–10) every 4 hours for a total of 10 times. The pain score was routinely assessed for all postoperative patients in my clinic. The pain scores were analyzed to determine their correlations with the total volume of residual carbon dioxide gas. The total volume of residual carbon dioxide gas was correlated with operative wound pain scores of 3, 4, 5, 6, 7, 8, 9, and 10 (Pearson correlation = −0.114, *P* = .048; −0.140, .015; −0.185, .001; −0.218, .000; −0.163, .005; −0.231, .000; −0.217, .000; and −0.230, .000, respectively). However, the total volume of residual carbon dioxide was correlated only with a shoulder pain score of 2 (Pearson correlation = 0.230, *P* = .043). The postoperative shoulder pain scores were analyzed to determine the correlation with operative wound pain scores. The postoperative shoulder pain scores were not correlated with the operative wound pain scores except for the pain score of 10 (Pearson correlation = 0.233, *P* = .041). With this, postoperative wound pain and postoperative shoulder pain may have other causes.

In particular, the shoulder pain reported at the time of the chest X-ray was not correlated with the total volume of residual gas. Hence, the total volume of residual carbon dioxide gas was more correlated with the operative wound pain score than the shoulder pain score.

Next, clinical characteristics and operative outcomes were analyzed according to the use of a drainage tube. Among the patients enrolled in this study, a drainage tube was not used in the 1-port group but was used in the 2- and 3-port groups. There were no significant differences in age, parity, height, weight, BMI, operative time, postoperative WBC count, change in Hb level, or the time to the first passage of gas between the groups with and without a drainage tube. However, there were significant differences in the preoperative WBC, postoperative hospitalization duration, right residual carbon dioxide volume, left residual carbon dioxide volume, total residual carbon dioxide volume, and shoulder pain score between the groups.

Because there were more cases of inflammatory diseases, such as tubo-ovarian abscesses or pelvic inflammatory diseases, among patients with a drainage tube, the preoperative WBC count was higher in patients with a drainage tube than in patients without (Table 4). While the postoperative WBC count was not significantly different between these groups, it was lower in patients with a drainage tube, suggesting an improvement in inflammation after the operation and antibiotic treatment. Almost all cases with inflammatory diseases were included in the 2- or 3-port group, and this condition required longer hospital stays for antibiotic treatments. However, the 1-port group had no cases of inflammatory diseases. With this, the postoperative hospitalization duration of the 2- or 3-port group was shown to be longer than that of the 1-port group.

**Table 4:** Clinical characteristics and operative outcomes according to the use of a drain tube.

	Without drain tube (N = 199)	With drain tube (N = 127)	*P* value
Age (yr)	34.7 ± 12.1	34.6 ± 12.1	.962
Parity	1.1 ± 1.2	1.0 ± 1.1	.400
Height (cm)	159.6 ± 5.8	160.1 ± 6.5	.475
Weight (kg)	59.8 ± 11.7	59.4 ± 13.3	.777
BMI (kg/m^2^)	23.5 ± 4.3	24.2 ± 13.2	.453
Operative time (min)	78.8 ± 21.4	75.3 ± 24.6	.176
Preoperative WBC	6347.2 ± 1926.9	9218.1 ± 4773.2	.000*
Postoperative WBC	6860.3 ± 1804.3	6552.0 ± 2264.7	.175
Hb change (g/dL)	1.7 ± 0.8	1.9 ± 1.2	.054
Time to first passage of gas (h)	43.7 ± 16.3	40.8 ± 16.7	.132
Postoperative hospitalization duration (days)	3.9 ± 0.7	4.5 ± 1.1	.000*
Rt. residual carbon dioxide volume (mL)	72.6 ± 98.2	16.3 ± 64.6	.000*
Lt. residual carbon dioxide volume (mL)	84.0 ± 135.9	7.6 ± 29.6	.000*
Total residual carbon dioxide volume (mL)	156.5 ± 179.1	24.0 ± 88.2	.000*
Shoulder pain score	2.1 ± 1.9	1.3 ± 1.0	.031*

BMI = body mass index, WBC = white blood cell.

* *P* < .05, significant.

The postoperative hospitalization duration was longer for patients with a drainage tube than for those without a drainage tube because it was shorter in the 1-port group than in the 2- and 3-port groups. The right, left, and total residual carbon dioxide gas volumes and the shoulder pain score were lower in patients with a drainage tube than in patients without a drainage tube. No patient with a drainage tube was readmitted for the management of inflammation or fever after postoperative care. Therefore, the use of a drainage tube played a role in decreasing the volume of residual carbon dioxide gas and lowering the pain scores without increasing the risk of postoperative infection.

## 4. Discussion

The origin of the referred pain reported by patients in the shoulder after laparoscopy is only partly understood. One theory proposes that tissue trauma is caused by stretching of the peritoneum and diaphragm secondary to the pneumoperitoneum. Residual carbon dioxide gas can also irritate the phrenic nerve, resulting in postoperative shoulder pain and upper abdominal pain.^[13]^ Another theory is based on the finding that pockets of residual CO_2_ gas are left between the liver and the diaphragm after surgery. The presence of gas pockets between the liver and diaphragm may lead to referred pain in the shoulder.^[14]^ A third theory regarding this referred shoulder pain suggests that CO_2_ gas acts as an irritant. This theory is based on the assumption that CO_2_ gas is converted to carbonic acid on the moist surface of the peritoneum, irritating the peritoneum and diaphragm and leading to referred pain in the shoulder.^[13]^

To decrease postoperative shoulder pain, some authors have used deep neuromuscular blockade and low-pressure pneumoperitoneum.^[15]^ In a randomized controlled trial, deep neuromuscular blockade and low-pressure pneumoperitoneum (8 mm Hg) resulted in a lower incidence of shoulder pain after laparoscopic hysterectomy than moderate neuromuscular blockage and standard-pressure pneumoperitoneum (12 mm Hg). In a retrospective case cross-sectional study, abdominal compression and pulmonary recruitment maneuvers after transvaginal natural orifice transluminal endoscopic surgery (vNOTES) might be considered to decrease postlaparoscopic shoulder pain.^[16]^ In a meta-analysis of randomized controlled trials, the authors concluded that a pulmonary recruitment maneuver with 40 cm H_2_O performed either alone or accompanied by intraperitoneal saline was a promising intervention for alleviating shoulder pain within 48 hours following gynecologic laparoscopy.^[17]^ With these articles, it was suggested that free pneumoperitoneum is the cause of postoperative shoulder pain and that methods to decrease free air were a strategy for postoperative shoulder pain.

A study of 20 patients who underwent gynecological laparoscopic surgery revealed a correlation between the residual intraperitoneal gas volume and pain scores. Pain scores are subjective parameters, while the volume of residual intraperitoneal air is more objective. Lee et al^[18]^ investigated whether active suction decreases residual intraperitoneal gas and whether residual gas decreases pain scores. They performed a prospective randomized controlled study to investigate whether active gas suction reduces the intraperitoneal residual carbon dioxide volume and analyzed the effect of active gas suction on postoperative pain after laparoscopic cholecystectomy.^[18]^ They found that active suction significantly decreased the residual intraperitoneal gas volume and postoperative pain after laparoscopic surgery. In another randomized controlled study, the effect of active gas aspiration on postoperative shoulder pain relief after diagnostic laparoscopy was significantly superior to that of simple gas evacuation and was not associated with any adverse events.^[19]^ In our study, although the shoulder pain score was not correlated with the volume of residual carbon dioxide, a drainage tube may play a similar role in decreasing the volume of residual carbon dioxide gas. However, the abdominal pain score was correlated with the volume of residual carbon dioxide. Although the shoulder pain score is a subjective parameter, the correlation between the residual gas volume and the shoulder pain score has been supported by clinical studies.

Drainage for peritoneal suction was investigated by Haghoo et al,^[20]^ who suggested that it may be useful for preventing postoperative shoulder pain among patients undergoing gynecological laparoscopic surgery and could decrease the need for pain medication.^[21]^ In contrast, Kandil and El Hefnawy^[13]^ suggested that the origin of pain after laparoscopic cholecystectomy is multifactorial. Because patients with and without drainage tubes have a similar incidence of postoperative shoulder pain, drains should not be used with the intention of preventing shoulder pain. In a prospective randomized study, the authors suggested that prophylactic surgical drainage may not be necessary to prevent postoperative morbidity after laparoscopically assisted vaginal hysterectomy.^[22]^ In a meta-analysis, abdominal pain 24 hours after surgery was less severe in the no-drain group. No significant difference was present with respect to the presence and quantity of subhepatic fluid collection, shoulder tip pain, parenteral ketorolac consumption, nausea, vomiting, or hospital stay. With this, the authors concluded that the study was unable to prove that drains were useful in reducing complications in laparoscopic cholecystectomy.^[23]^

However, drains still play a role in gynecological laparoscopy in select women, such as those with persistent discharge from raw surfaces, bowel injury, or frank pus in the abdomen. In our study, the use of a drainage tube decreased the volume of residual carbon dioxide gas and had no adverse effects on postoperative inflammation. Because there was a greater incidence of inflammatory disease among patients with a drainage tube, such as tubo-ovarian abscesses or pelvic inflammatory diseases, the preoperative WBC count was higher in patients with a drainage tube than in patients without. Although the postoperative WBC count was not significantly different between these groups, it was lower in patients with a drainage tube, suggesting an improvement in inflammation after the operation and antibiotic treatment.

Our study has certain limitations. First, postoperative shoulder and abdominal pain were evaluated using a pain score based on a VRS. Because the pain score is a subjective parameter, it has limited value in providing an objective quantification of pain. It is possible that the low correlation between shoulder pain and the volume of residual carbon dioxide gas was due to this limitation.

Second, in our study, none of the patients who underwent 1-port laparoscopy had a drainage tube because of the possibility of umbilical operative wound problems. The effect of the drain tube was not further analyzed in ruling out the potential factors influencing the amount of residual carbon dioxide gas. With this, our analysis of drainage tube use has some limitations. Although the drainage tube may be a main factor in decreasing the amount of residual carbon dioxide gas, other factors were suggested, including the additional port, total amount of insufflated carbon dioxide gas, operation time, and other factors that would be influenced by the amount of residual carbon dioxide gas.

Third, because there were fewer enrolled patients in the 3-port group, the comparisons of clinical outcomes between the 2- and 3-port laparoscopy groups showed no significant differences. The study period of the retrospective cohort study was identified as the period during which 1 surgeon had performed laparoscopic adnexal surgeries in the hospital. For this reason, despite the small size of the study groups, bias resulting from the surgeon or medical treatment patterns can be excluded.

Fourth, the enrolled patient study group was consecutive and was divided into 3 study groups according to the number of ports used. Propensity score matching was not performed.

## 5. Conclusions

A retrospective cohort study was performed via a review of the medical records and radiological studies related to patients who underwent laparoscopic adnexal surgery between March 2005 and June 2018. The enrolled patients were divided into 3 study groups according to the number of ports used into the 1-port (198 patients), 2-port (114 patients), and 3-port (14 patients) groups.

The total volume of postoperative residual carbon dioxide gas was significantly different between the 1- and 2-port groups and the 1- and 3-port groups. However, the difference between the 2- and 3-port groups was not significant. A drainage tube was used in all cases of 2-port and 3-port laparoscopy and played a major role in decreasing the volume of postoperative residual carbon dioxide gas. Clinical characteristics and operative outcomes were analyzed according to the use of a drainage tube. There were significant differences in the preoperative WBC, postoperative hospitalization duration, right residual carbon dioxide volume, left residual carbon dioxide volume, total residual carbon dioxide volume, and shoulder pain score between the groups with and without a drainage tube. Therefore, the use of a drainage tube played a role in decreasing the volume of residual carbon dioxide gas and lowering the pain scores without increasing the risk of postoperative infection.

Although we found that the residual gas volume was less correlated with the shoulder pain score than with the operative wound pain score, we also found that the volume of residual carbon dioxide gas and the shoulder pain score were lower in patients with a drainage tube than in patients without a drainage tube. Although the drainage effect on shoulder pain has been controversial, a drainage tube can be safely used to decrease the residual carbon dioxide gas volume and pain scores without increasing the risk of postoperative infection.

## Author contributions

Conceptualization: Sang Wook Yi.

Data collection: Sang Wook Yi.

Methodology: Sang Wook Yi.

Writing – original draft/review & editing: Sang Wook Yi.
